# Brain tumour post-treatment imaging and treatment-related complications

**DOI:** 10.1007/s13244-018-0661-y

**Published:** 2018-11-08

**Authors:** Alexander T. Kessler, Alok A. Bhatt

**Affiliations:** Department of Imaging Sciences, University of Rochester Medical Center, 601 Elmwood Avenue, P.O. Box 648, Rochester, NY 14642 USA

**Keywords:** Brain neoplasms, Glioma, Neoplasm metastasis, Radiotherapy, Review

## Abstract

**Purpose:**

The imaging of primary and metastatic brain tumours is very complex and relies heavily on advanced magnetic resonance imaging (MRI). Utilisation of these advanced imaging techniques is essential in helping clinicians determine tumour response after initiation of treatment. Many options are currently available to treat brain tumours, and each can significantly alter the brain tumour appearance on post-treatment imaging. In addition, there are several common and uncommon treatment-related complications that are important to identify on standard post-treatment imaging.

**Methods:**

This article provides a review of the various post-treatment-related imaging appearances of brain neoplasms, including a discussion of advanced MR imaging techniques available and treatment response criteria most commonly used in clinical practice. This article also provides a review of the multitude of treatment-related complications that can be identified on routine post-treatment imaging, with an emphasis on radiation-induced, chemotherapy-induced, and post-surgical entities.

**Summary/Conclusion:**

Although radiological evaluation of brain tumours after treatment can be quite challenging, knowledge of the various imaging techniques available can help the radiologist distinguish treatment response from tumour progression and has the potential to save patients from inappropriate alterations in treatment. In addition, knowledge of common post-treatment-related complications that can be identified on imaging can help the radiologist play a key role in preventing significant patient morbidity/mortality.

**Teaching points:**

• *Contrast enhancement does not reliably define tumour extent in many low-grade or infiltrative gliomas.*

• *Focal regions of elevated cerebral blood volume (rCBV) on dynamic susceptibility contrast (DSC) perfusion-weighted imaging are suggestive of tumour growth/recurrence.*

• *Brain tumour treatment response criteria rely on both imaging and clinical parameters.*

• *Chemotherapeutic agents can potentiate many forms of radiation-induced injury.*

• *Ipilimumab-induced hypophysitis results in transient diffuse enlargement of the pituitary gland.*

## Introduction

Primary and metastatic brain tumours are frequently encountered in the daily practice of neuroimaging. Many options are currently available to treat these neoplasms and revolve around a combination of surgery, radiation, and/or novel chemotherapeutic agents. These treatment choices are ever growing and each results in alterations in pathophysiology that can drastically change the imaging appearance of the tumour. The interpretation of post-treatment imaging has therefore become much more complex, most notably with high-grade gliomas where the combination of radiation and anti-neoangiogenesis drugs can result in either increasing or decreasing local enhancement, irrespective of tumour progression/regression. For this reason, it is imperative that radiologists have a thorough understanding of the advanced imaging techniques available to image these tumours as well as the possible common treatment-related complications. The purpose of this review is to briefly review brain tumour imaging techniques, discuss the most commonly used brain tumour treatment response criteria implemented in clinical practice, and illustrate the broad range of post-treatment-related complications that can be identified on routine post-treatment imaging.

## Brain tumour imaging techniques

**Fig. 1:**
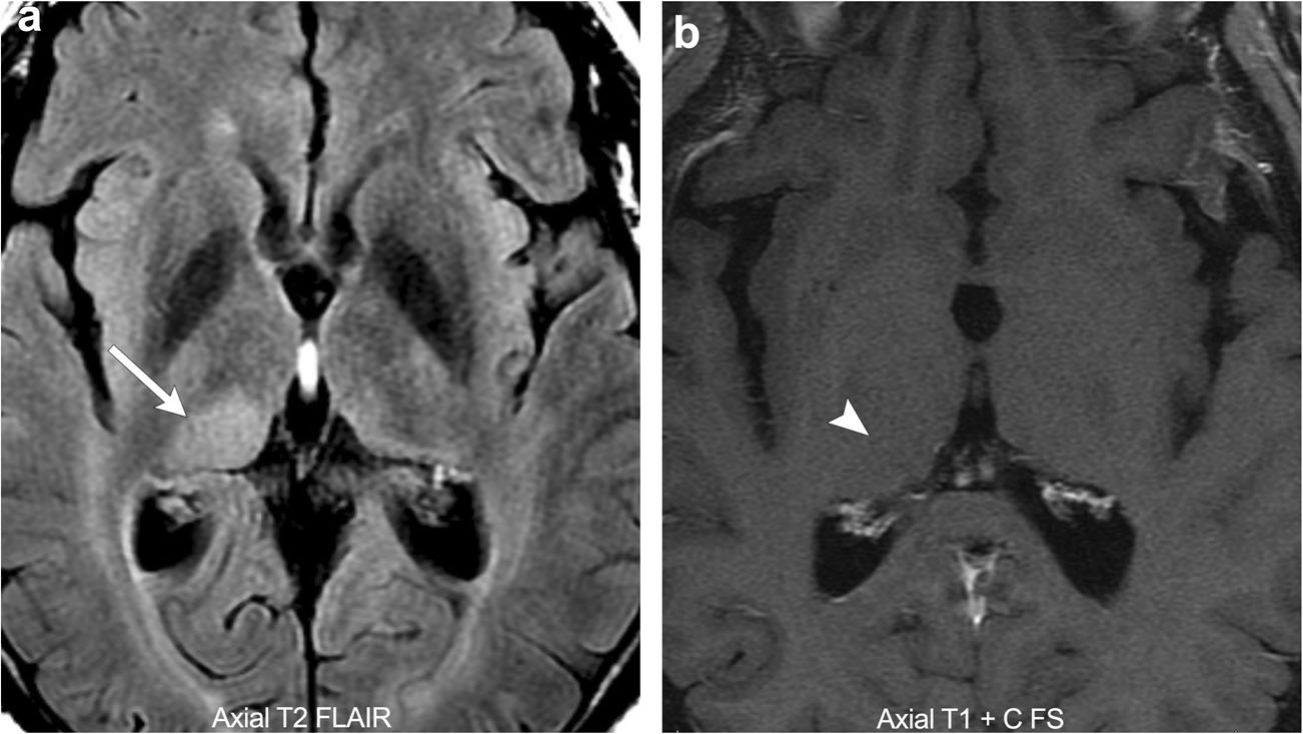
**Pathology-proven low-grade glioma**. A 58-year-old male with a T2 FLAIR hyperintense mass in the right thalamus (arrow). No associated enhancement (arrowhead)

Contrast-enhanced T1-weighted MRI imaging is the workhorse of brain tumour imaging. It is easy to perform and accurately depicts the margins of most brain metastases and dural-based lesions. However, regarding primary brain neoplasms, particularly gliomas, it is not as reliable because these tumours often demonstrate non-enhancing or infiltrative components (Fig. 1). In these cases, T2 fluid attenuated inversion recovery (FLAIR) imaging is often the sequence of choice as it clearly delineates abnormal signal from normal brain parenchyma. In fact, low-grade gliomas rarely exhibit vasogenic oedema, and therefore T2 FLAIR imaging can be particularly accurate in delineating tumour extent. However, with high-grade gliomas, T2 FLAIR imaging has its limitations in that it cannot reliably differentiate infiltrating tumour from vasogenic oedema as both are hyperintense on T2 FLAIR sequences. Therefore, we often rely on advanced imaging techniques to further differentiate residual/recurrent tumour from post-treatment changes. Of these, the most common advanced imaging techniques include: diffusion-weighted imaging (DWI), perfusion-weighted imaging (PWI), and MR spectroscopy. Individually, none of these techniques have proven very specific; however, a thoughtful synthesis using all of them can usually allow the radiologist to correctly separate tumour from post-treatment changes.

### Diffusion-weighted imaging

DWI provides a visual and quantitative representation of the diffusivity of water via creation of a DWI map (typical b value of 1000) and an apparent diffusion coefficient (ADC) map. Many pathophysiological processes result in diffusion restriction; however, in the context of brain tumour imaging the most common aetiologies include abnormally high cellularity, cellular injury, and peritumoural oedema. Highly cellular tumours, such as lymphoma and many high-grade gliomas, result in decreased water diffusivity through a relative reduction in the extracellular space for protons to move about [1]. This results in low ADC signal and some studies have shown that glioma grade often inversely correlates with the minimum ADC value identified within the tumour [2]. Knowledge of a tumour’s baseline cellularity is very helpful as any new area of low ADC signal on follow-up imaging should raise the suspicion for tumour recurrence/progression.

**Fig. 2:**
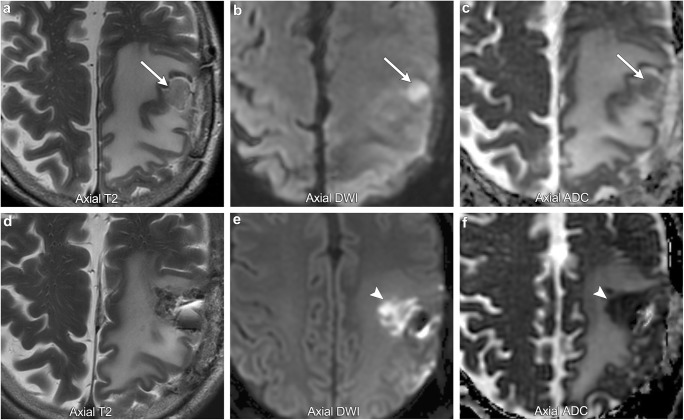
**Postoperative ischaemia**. A 39-year-old male with preoperative imaging (first row of images) demonstrating a T2 hypointense, diffusion-restricting left parietal lobe mass, suggestive of a hypercellular tumour (arrows). Immediate postoperative imaging (second row of images) shows Gliadel wafers in the resection cavity and a new area of diffusion restriction medial to the resection cavity suggestive of postoperative ischaemia (arrowheads)

Diffusion restriction in the setting of cellular injury has been well described in the literature in the setting of vascular infarction. In the postoperative setting, cellular injury is also very common with one study demonstrating new diffusion restriction surrounding the surgical cavity in 64% of resected gliomas, of which 93% went on to develop encephalomalacia on serial imaging, suggesting cellular injury (Fig. 2) [3]. In the postoperative setting, cellular injury is multifactorial, most commonly due to direct surgical trauma, vascular injury, and devascularisation of tumour [1]. In all cases, diffusion restriction occurs because of cellular injury resulting in acute intracellular swelling, which in turn decreases the extracellular space for protons to move about. Like vascular infarction, post-treatment cytotoxic oedema often demonstrates a phase of contrast enhancement in the subacute phase that resolves on follow-up imaging as encephalomalacia forms. It is therefore very important to correlate any new enhancement with the immediate postoperative DWI so as not to erroneously diagnose tumour progression when it is actually postoperative injury.

Peritumoural oedema refers to the area of abnormal signal surrounding the enhancing component of a brain tumour. The problem with this term is that it encompasses two separate pathophysiological processes (vasogenic and infiltrative oedema) that have very different implications for treatment. In vasogenic oedema, there is increased fluid in the extracellular space due to alterations in vascular permeability resulting in leakage of plasma fluid and protein. This commonly occurs with metastatic lesions and non-infiltrating primary brain tumours and is typically reversible. It classically does not result in diffusion restriction and the area of abnormal signal is not considered to be within the margins of the tumour. The major clinical implication of vasogenic oedema is in regard to any mass effect it creates on adjacent normal structures. In infiltrative oedema, there is both perivascular infiltration of tumour and leakage of fluid into the extracellular space. This commonly occurs with higher grade infiltrating gliomas and makes it difficult to accurately define tumour margins. Theoretically, perivascular infiltration of tumour should decrease water diffusivity producing low ADC signal. However, many studies have shown that the areas of tumour infiltration are quite small and DWI is not very reliable for differentiating vasogenic from infiltrative oedema [4]. In these cases, DWI must be combined with other advanced imaging techniques so that accurate tumour margins can be identified and treatment can be planned accordingly.

### Perfusion-weighted imaging

PWI is a noninvasive imaging technique for measuring brain tumour vascularity. It indirectly provides information on tumour angiogenesis and altered capillary permeability, both of which are present in many types of brain tumours. This is of particular importance on post-treatment imaging, as areas of increased perfusion can be suggestive of tumour growth or recurrence. The most commonly used perfusion techniques include dynamic susceptibility contrast (DSC), dynamic contrast-enhanced (DCE), and arterial spin labelling (ASL) MR imaging.

**Fig. 3:**
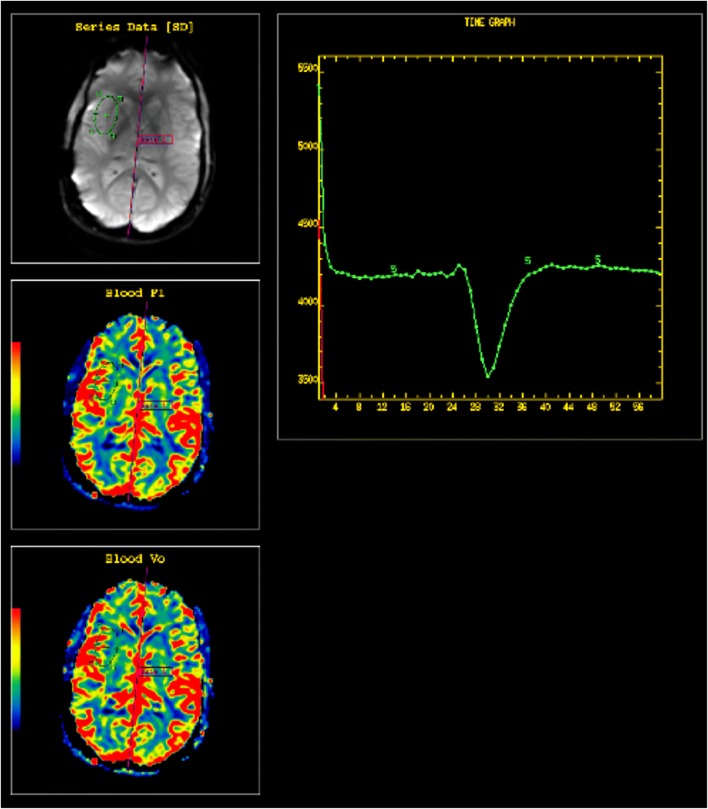
**Normal DSC perfusion maps** (CBF and CBV) with an adequate time graph

**Fig. 4:**
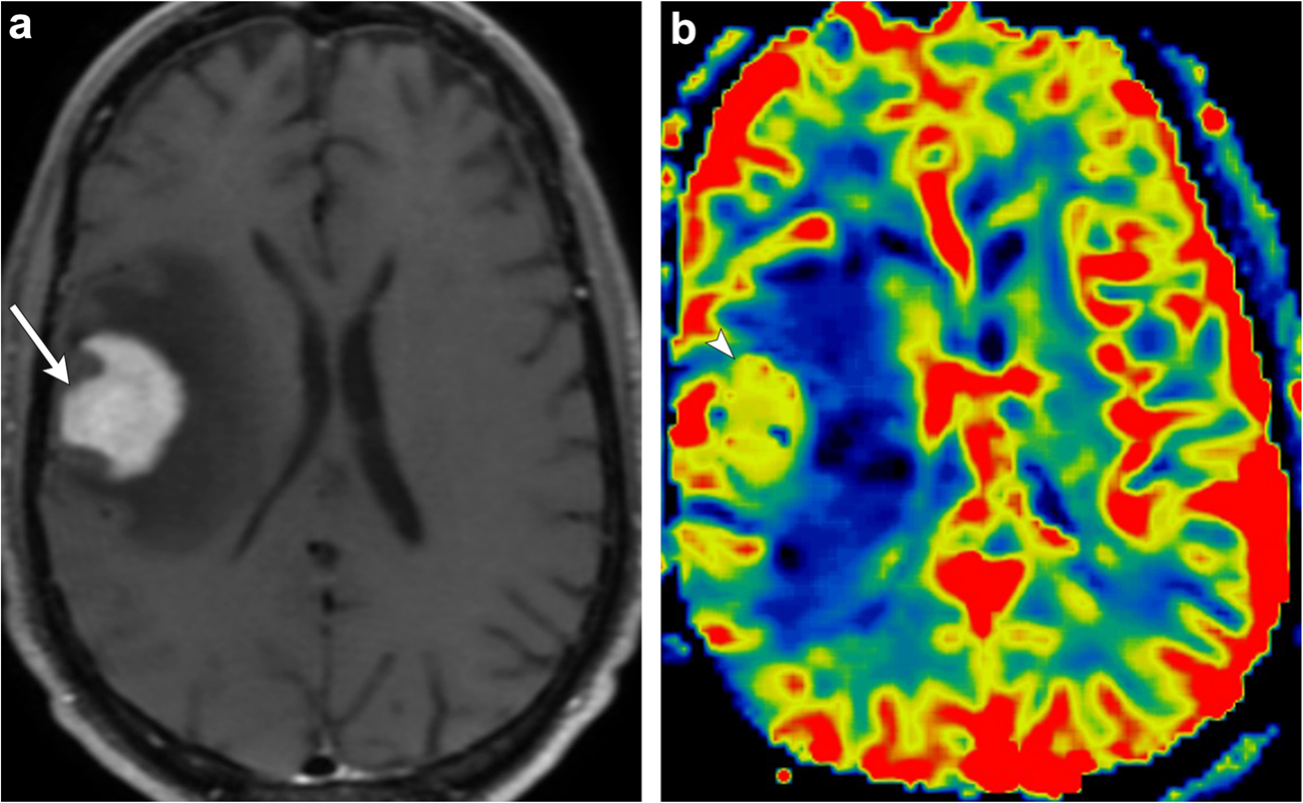
**Pathology-proven CNS lymphoma**. A 67-year-old male with an avidly enhancing right frontal lobe mass (arrow). Colour perfusion map demonstrates elevated rCBV within the tumour (arrowhead)

**Fig. 5:**
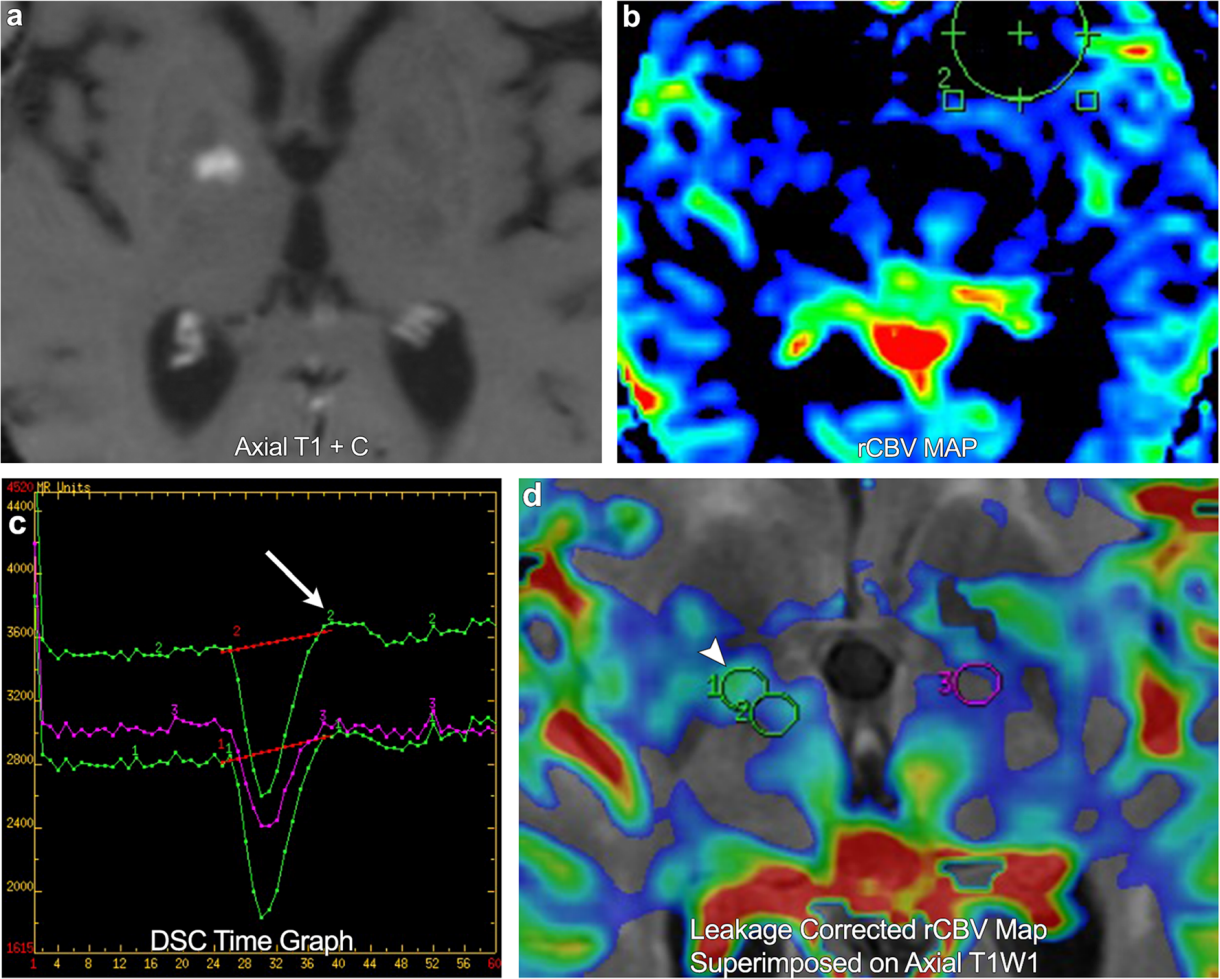
**A 73-year-old female with right frontal lobe glioblastoma multiforme**. Post-treatment imaging demonstrates a new region of enhancement in the right basal ganglia. DSC rCBV map shows no definite increased perfusion. However, the time graph demonstrates overshoot (arrow). DSC leakage-corrected map superimposed on axial T1WI now shows asymmetric elevated rCBV in the enhancing lesion (arrowhead), compatible with a new site of tumour

DSC perfusion imaging measures perfusion by assessing susceptibility-induced T2* signal loss from the bolus of contrast as it passes through the capillary bed. This loss of signal is depicted as a signal intensity-time curve; the area under the curve is then calculated to derive the relative cerebral blood volume (rCBV) (Fig. 3). Many brain tumours demonstrate inherent hypervascularity and associated elevated rCBV. The most frequently encountered of these include the high-grade astrocytomas, oligodendrogliomas, choroid plexus tumours, meningiomas, and CNS lymphoma (Fig. 4). It is therefore important to recognise any new area of elevated rCBV on posttreatment imaging of these tumours, as this may be a marker for tumour growth/recurrence. A pitfall of DSC perfusion is that rCBV is calculated with the underlying assumption that there is no contrast leakage or recirculation. However, in reality there is always some degree of contrast leakage due to brain tumour violation of the blood-brain barrier. Visually, this can be seen on the DSC time curve as “overshooting”, where the signal returns to a higher point than baseline after clearance of the contrast bolus. This is important to recognise because it can erroneously underestimate perfusion within the area of leakage (Fig. 5). Numerous methods exist to attempt to correct for contrast leakage and are widely used in clinical practice. Common methods include pre-injecting a small contrast bolus to saturate the T1 signal in local tissues using dual-echo pulse sequences and baseline subtraction techniques [5, 6].

DCE perfusion imaging involves performing repeated T1-weighted imaging after contrast administration to produce a contrast signal time intensity curve. These data can then be analysed to calculate multiple parameters reflective of tumour vascularity. The most commonly calculated parameter is that of the transfer coefficient k^trans^, which represents the degree of permeability between the plasma and extravascular extracellular space [7]. It is important to note that when the blood-brain barrier is intact, k^trans^ more truly reflects vascular permeability; however, when the blood-brain barrier is disrupted by tumour, k^trans^ becomes more representative of cerebral blood flow [8]. Although still under investigation, many authors suggest that alterations in the k^trans^ of brain tumours likely occur because of the formation of immature hyperpermeable vessels, a common occurrence in growing/progression of brain tumours. Therefore, as with rCBV in DSC perfusion imaging, elevations in k^trans^ may also be a potential marker for tumour growth/recurrence [9].

ASL perfusion imaging is a non-contrast perfusion technique that uses inversion pulses to magnetically label water protons in inflowing arterial blood. By comparing the difference in signal between labelled images and non-labelled images, cerebral blood flow (CBF) can then be quantified. This technique has been widely established as a useful technique in assessing cerebral perfusion in stroke and dementia; however, more recent literature has demonstrated growing utility in brain tumour imaging. Although ASL imaging does not directly measure tumour blood volume, many studies have shown that blood volume and blood flow are strongly correlated, and therefore focal areas of elevated CBF on ASL imaging may be a marker for tumour growth/recurrence. Compared with DSC perfusion techniques, ASL has the added advantage of a higher signal-to-noise ratio (SNR) and no need for intravenous contrast. However, major limitations include longer scan time and lower spatial resolution compared with DSC perfusion imaging [10].

### MR spectroscopy

**Fig. 6:**
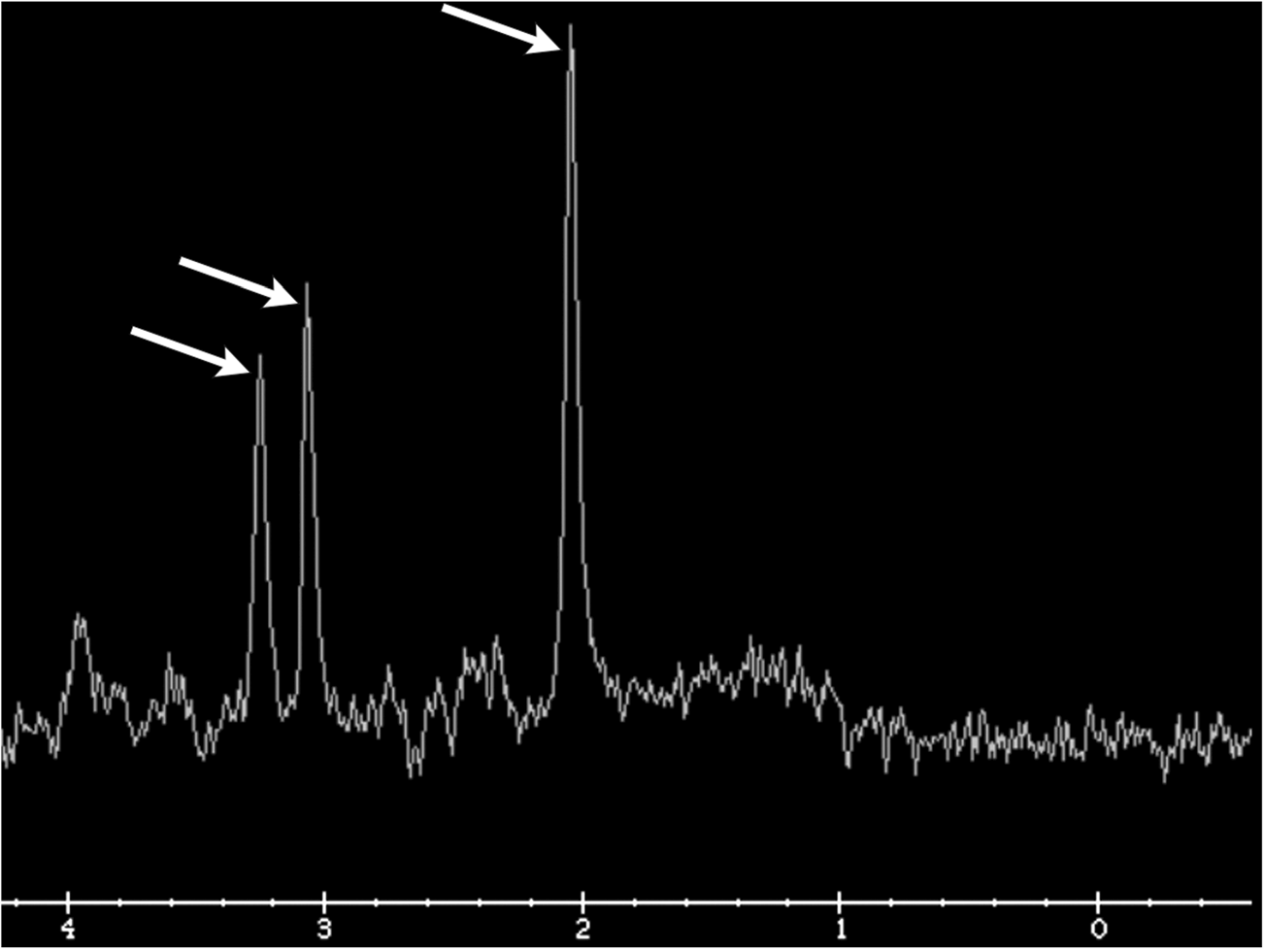
**Hunter’s angle**. Normal MR spectroscopy demonstrating a gradual increase in metabolite peaks of choline, creatine, and NAA (arrows). No lipid/lactate peak is present

**Fig. 7:**
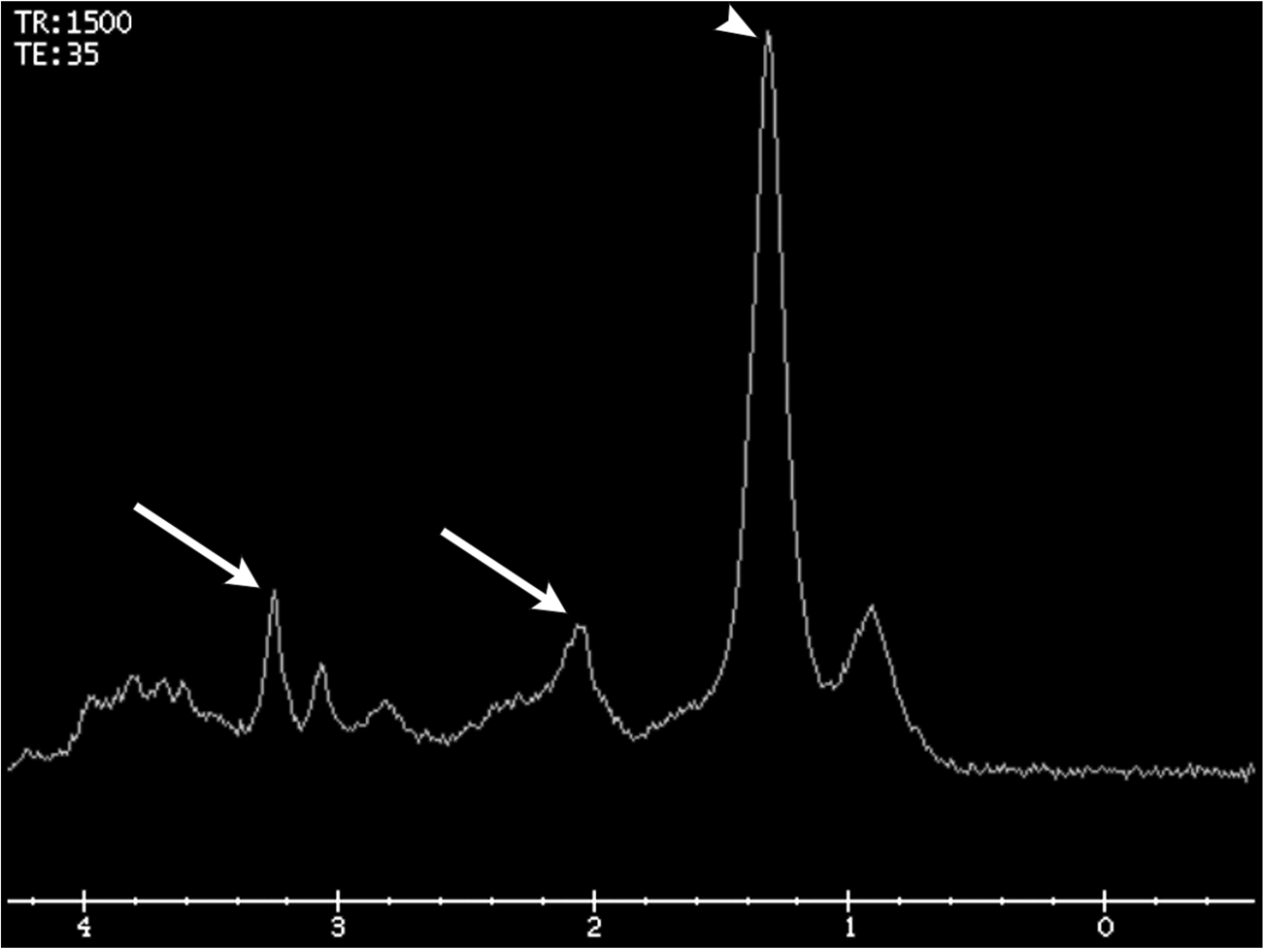
**MR spectroscopy of pathology-proven radiation necrosis**. Decreased choline, creatine, and NAA peaks (arrows) and an elevated lipid/lactate peak (arrowhead)

^1^H-MR spectroscopy is an MRI technique that creates a quantitative representation of the distribution of metabolites within a specified region of brain tissue. This can be performed via a single- or multivoxel acquisition and is often used to better characterise areas of abnormal brain tissue. The normal spectrum of brain metabolites typically shows relative elevation of choline, creatine, and N-acetylaspartate (NAA) in relation to the other metabolites, reflecting the normal composition of brain tissue. These three metabolite peaks show a normal gradual increase in concentration from left to right, creating the so-called “Hunter’s angle” (Fig. 6). Although there is much debate in the literature, many studies suggest that specific changes in metabolite patterns can be seen with different types of brain tumours. For example, primary gliomas typically demonstrate elevated lipid, lactate, choline, and myoinositol peaks and a decreased NAA peak [11]. In posttreatment imaging, ^1^H-MR spectroscopy is most helpful in differentiating focal areas of radiation necrosis from residual/recurrent tumour. The cellular damage that occurs in radiation necrosis classically results in decreased NAA, choline, and creatine peaks and an elevated lipid/lactate peak (Fig. 7). Interestingly, it is important to note that early stage radionecrosis may actually demonstrate elevation of the choline peak. That being said, in real clinical practice, areas of suspected radiation injury are usually comprised of a mixture of tumour cells and radiation necrosis [12]. This makes interpretation quite difficult and many authors suggest that ^1^H-MR spectroscopy cannot reliably differentiate the two in most cases [13].

### Future advanced imaging techniques

It is important to note that alternative brain tumour imaging techniques, such as chemical exchange saturation transfer (CEST) and sodium MR imaging, have been increasingly described in the literature. These techniques are primarily in the research stage but seem to show promise in better characterising tumours post treatment. For these reasons a discussion on brain tumour imaging cannot be complete without at least mentioning these techniques. CEST imaging is a novel form of MR imaging in which a radiofrequency pulse is applied resulting in saturation of a particular chemical species. Over time, this magnetic saturation is transferred to water molecules via an exchange of protons, resulting in a decrease in water signal. This decrease in water signal can then be detected and, from this, an indirect measurement of the originally saturated species can be obtained. Although many chemical species are currently being investigated, Park et al. have shown that CEST amide proton transfer (APT) imaging can reliably distinguish tumour progression from a treatment-related effect as well as improve the diagnostic accuracy when combined with conventional perfusion-weighted and contrast-enhanced T1-weighted imaging techniques [14]. Sodium MR imaging is another novel MRI technique in which the magnetic moments of ^23^Na nuclei, instead of traditional ^1^H nuclei, are used to create image contrast. Sodium nuclei are extremely abundant throughout the human body and have been found to yield the second strongest nuclear magnetic resonance (NMR) signal after ^1^H nuclei [15]. In post-treatment brain tumour imaging, this is particularly useful as elevations in the intracellular tissue sodium concentration (TSC) are thought to be directly related to an alteration in Na+/K+ pump exchange and cell death. Therefore, some authors suggest that sodium MR imaging performed during radiation treatment can be used to actively monitor the spatial distribution of tissue response and therefore allow for individualised changes in a given patient’s treatment algorithm [16].

Although these imaging techniques appear to demonstrate clinical utility, more research is needed to establish whether these techniques can be routinely used for post-treatment imaging.

## Treatment response criteria

It is imperative that radiologists be familiar with the treatment response criteria that referring clinicians are using to assess patients with brain tumours. Many of these algorithms rely heavily on imaging and therefore radiologists play an important role in dictating clinical management. Although each brain tumour behaves differently and carries different histological grades/aggressiveness, it is helpful to focus on the two main categories of brain tumours: high-grade gliomas and metastases.

### High-grade glioma treatment response criteria

Historically, multiple different criteria were used to the assess treatment response of high-grade gliomas. The Levin criteria relied on qualitative changes in perilesional mass effect to ascribe treatment response [17]. The World Health Organization (WHO) Oncology Response Criteria relied on quantitative changes in tumour size to ascribe treatment response [18]. However, many soon realised that both of these criteria were only characterising the visual extent of the tumour and not accounting for biological features that were being seen clinically. In 1990, the Macdonald criteria were introduced to address this by adding two clinical parameters that were found to correlate with treatment response. These included: (1) decreased need for steroids and (2) stable or improved clinical status [19]. Although better than prior criteria, the imaging component of the Macdonald criteria had a major weakness in that it relied solely on measuring changes in the enhancing portions of the tumour. As previously discussed, using post-contrast sequences alone is not an accurate way to measure the extent of many high-grade gliomas because of their propensity for infiltrative disease, which may not have enhancement. Therefore, in 2010 the Response Assessment in Neuro-Oncology (RANO) criteria were released emphasising the importance of T2 FLAIR sequences in assessing non-enhancing components of infiltrative lesions. In fact, any significant increase in T2 FLAIR non-enhancing lesions while on a stable or increasing dose of corticosteroids now met criteria for disease progression [20]. Although other criteria have been published over the last several years (i.e., AVAglio and RTOG 0825), the RANO criteria are currently the most widely used in clinical practice [21].

In addition to the RANO criteria, two radiological phenomena have been well described in the literature and require a further in-depth discussion. These include pseudoprogression and pseudoresponse.

**Fig. 8:**
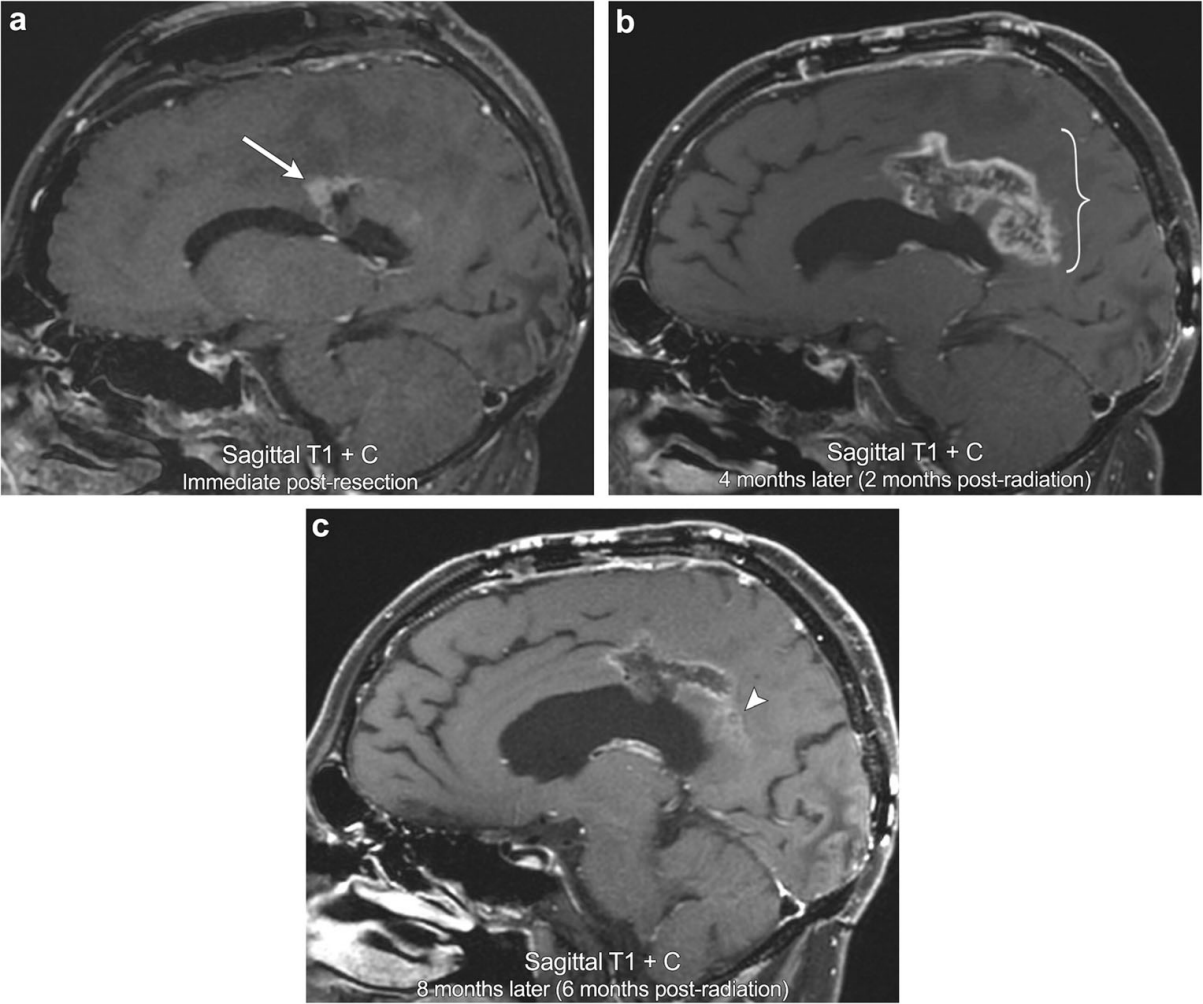
**Pseudoprogression**. A 46-year-old male with left callosal/pericallosal glioblastoma multiforme. Initial post-resection MRI demonstrates a small area of irregular enhancement along the anterior margin of the resection cavity (arrow). Follow-up imaging obtained 4 months later (2 months after completion of radiation) demonstrates a significant increase in peripheral, now thick, fluffy enhancement along the resection cavity, most marked posteriorly (bracket). Repeat MRI obtained 8 months post resection demonstrates decreased enhancement (arrowhead). No appreciable elevated perfusion or diffusion restriction was present (not shown). The constellation of findings is compatible with pseudoprogression on the 4-month postoperative scan

Pseudoprogression refers to the development of new or enlarging enhancement within the vicinity of a recently treated brain tumour. This imaging appearance initially mimics tumour progression, but should improve or stabilise on follow-up imaging [22]. This phenomenon is classically described in high-grade gliomas after initiation of treatment with chemoradiation, most commonly with temozolomide (TMZ) and radiation. Although poorly understood, the mechanism through which this occurs is thought to be due to chemoradiation inciting local inflammation, oedema, and a transient increase in the permeability of the blood-brain barrier resulting in regional hyperenhancement [23]. On imaging, this typically appears as thick, fluffy enhancement along the periphery of the lesion, which has higher ADC signal and lower rCBV compared with regions of viable tumour (Fig. 8). Pseudoprogression has been reported to occur in up to 21% of gliomas treated with TMZ chemoradiation and typically occurs within 2 months of treatment, earlier than the typical time period during which radiation necrosis occurs with radiation alone [24]. In addition, it is important to note that high-grade gliomas that express methylation of the O6-methylguanine DNA methyltransferase (MGMT) promoter typically show more frequent pseudoprogression than tumours that are unmethylated. In fact, a study by Balana et al. demonstrated that of 256 patients with GBM, those that expressed MGMT methylation had a 3.5-fold increase of developing pseudoprogression compared with true progression [25]. In addition, many MGMT methylated patients tend to also have better overall outcomes consisting of improved 2-year survival and increased time to recurrence [26]. Not only does this have important clinical implications for patient survival, but it also underscores that knowledge of a tumour’s MGMT methylation status can be extremely helpful for image interpretation in distinguishing pseudoprogression from true progression.

**Fig. 9:**
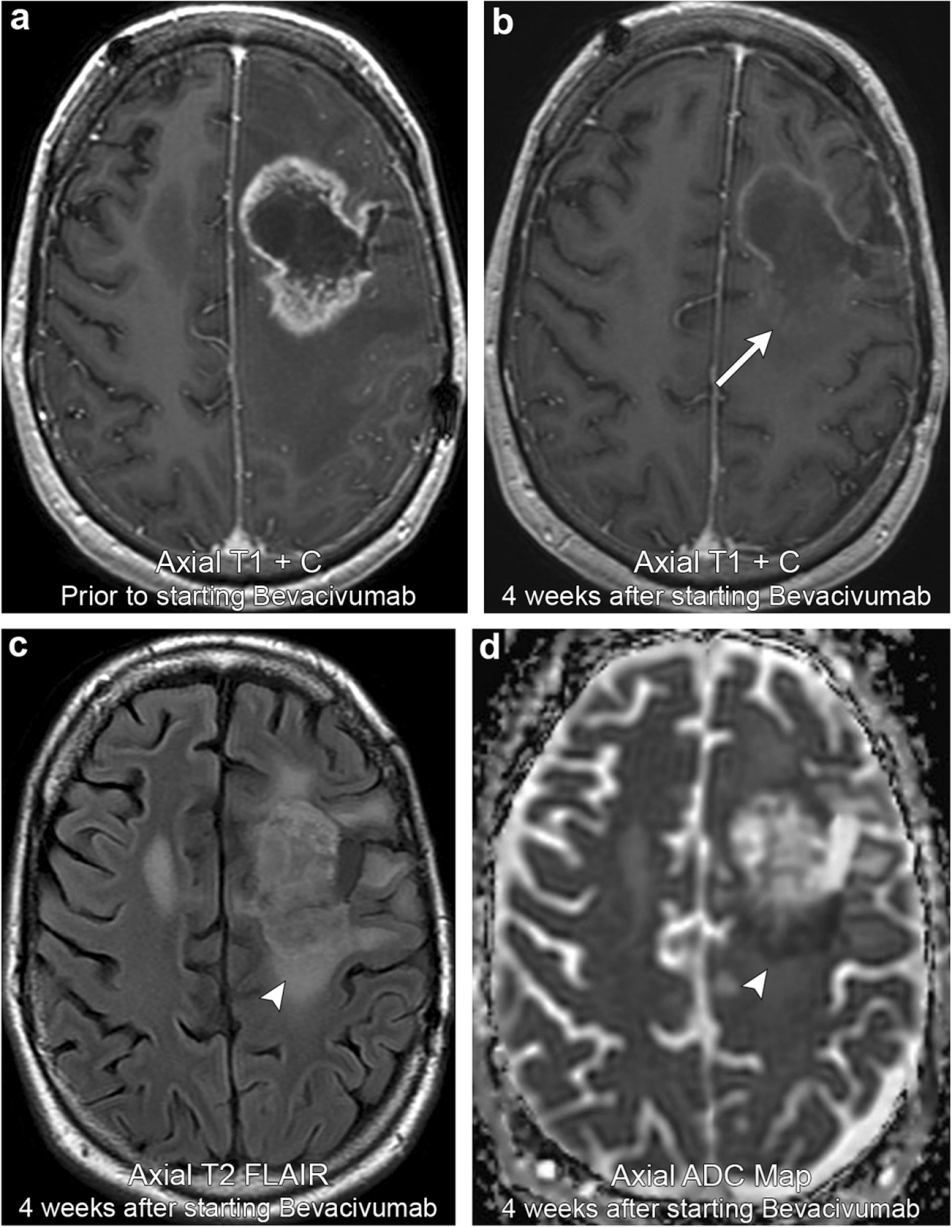
**Pseudoresponse**. A 38-year-old male with left frontal astrocytoma. Initial imaging demonstrates a peripherally enhancing lesion in the left frontal lobe. Follow-up imaging 4 weeks after starting bevacivumab demonstrates a marked interval decrease in enhancement (arrow). However, there is persistent T2 FLAIR hyperintensity and low ADC signal (arrowheads). Serial imaging afterwards (not shown) demonstrated progression in T2 FLAIR hyperintensity and mass effect, confirming pseudoresponse

Pseudoresponse refers to a rapid transient decrease in enhancement within a recently treated brain tumour. This imaging appearance initially mimics treatment response but careful inspection of the tumour often shows persistent T2 FLAIR hyperintensity and low ADC signal that worsens on follow-up imaging (Fig. 9). This decrease in enhancement can occur quite rapidly with one study suggesting that a relative decrease in tumour vessel size occurs as early as day 1 of treatment. However, this same study suggests that the majority of these patients demonstrate a reversal of vessel size towards abnormal values by day 56 of treatment [27]. This phenomenon is classically described in high-grade gliomas treated with antiangiogenic agents such as bevacizumab and cediranib that work through inhibition of vascular endothelial growth factor (VEGF); however, other therapeutic agents, such as steroids, have also been implicated. Many studies suggest that this decrease in enhancement is due to treatment-related alteration in vascular permeability via normalisation of leaky tumour vessels rather than true tumour reduction [22]. This is further supported by the fact that pseudoreponse is associated with a significant improvement in 6-month progression-free survival, but not overall survival [27]. It is therefore very important to be aware of which therapeutic agents have been administered when interpreting glioma post-treatment imaging so that an accurate distinction between pseudoresponse and true response can be made.

### Metastasis treatment response criteria

**Fig. 10:**
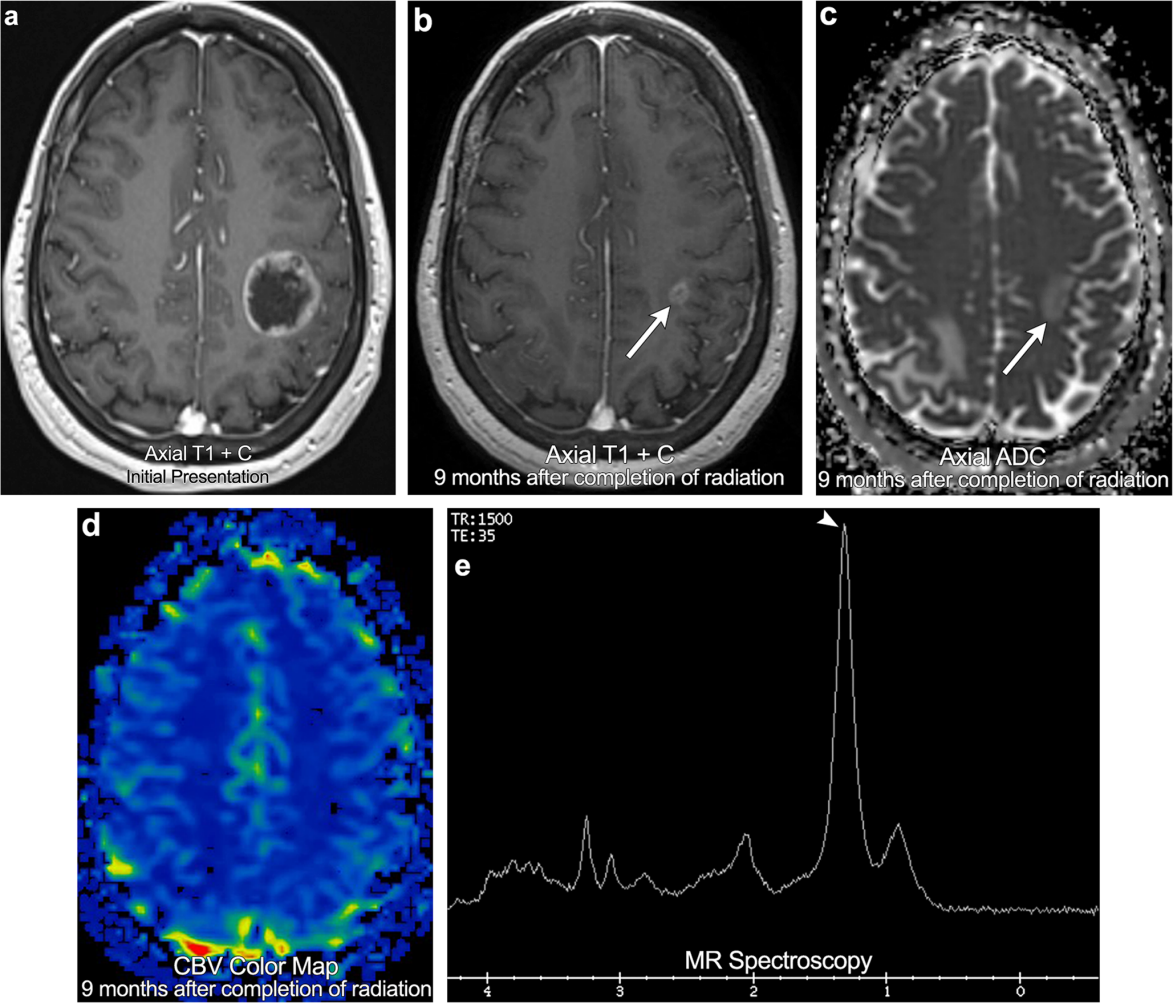
**Partial response**. A 55-year-old female with breast cancer. Initial staging MRI demonstrates a large rim-enhancing left frontoparietal brain metastasis. Follow-up imaging obtained 9 months after completion of radiation (not on steroid medication) demonstrates a > 30% decrease in the size of the metastasis, no associated low ADC signal, and the absence of elevated cerebral perfusion (arrows). Spectroscopy demonstrates suppressed NAA, choline, and creatine peaks as well as an elevated lipid/lactate peak (arrowhead). Findings consistent with partial response

Metastases are the most common cause for CNS malignancy in adults. Despite being so common, there is extensive variation in the literature regarding metastasis treatment and treatment response criteria. In fact, historically many clinical trials have actually excluded brain metastases from their inclusion criteria. In an effort to eliminate this confusion and standardise discussion of CNS metastases, in 2015 the Response Assessment in Neuro-Oncology Brain Metastases (RANO-BM) working group released the Response Assessment Criteria for Brain Metastases. These treatment criteria focus on objective measurements of tumour size as well as clinical criteria such as corticosteroid use and clinical deterioration [28]. These criteria rely heavily on medical imaging with radiological disease progression defined as a > 20% increase in lesion size or the presence of any new lesions. Partial response is defined as a ≥ 30% decrease in lesion size (Fig. 10), while stable disease encompasses everything in between. It is important to note that these criteria are limited to parenchymal brain metastases as leptomeningeal and calvarial metastases are often much more difficult to objectively measure and follow. Although CNS metastases are now starting to be included in more clinical trials, knowledge of the RANO-BM Response Assessment Criteria is essential to provide a framework to more effectively communicate with referring clinicians.

## Radiation-related complications

Radiation-induced brain injury encompasses a wide variety of both clinical and radiological findings that result from fractionated or whole-brain radiation. These injury patterns are often described as an expression of time from the initiation of radiation under the following categories: acute (days to weeks), early delayed (weeks to months), and late delayed (months to years).

Acute and early delayed radiation injury is thought to result from alterations in vascular permeability and disruption of the blood-brain barrier resulting in varying degrees of brain oedema/swelling. In contrast to late delayed injury, this alteration in physiology is usually reversible and resolves spontaneously without any histopathological correlate [29]. In the acute setting, patients typically present with vague signs related to increased brain oedema, most notably headache and drowsiness. However, it is important to note that on imaging, acute radiation injury may be difficult to identify as the radiation-induced brain oedema is often indistinguishable from the baseline vasogenic oedema incited by the brain tumour [22, 29]. In the early delayed setting, brain swelling may be accompanied by transient demyelination that often results in patients presenting with somnolence or attention/memory deficits. On imaging, this may appear as new areas of oedema or enhancement, typically within the vicinity of the irradiated tumour [29].

Late delayed radiation injury, on the other hand, is a much more serious form of injury due to a combination of vascular and glial injury resulting in irreversible and progressive white matter necrosis. The exact mechanism through which this occurs is not well understood but thought to be due to either vascular endothelial injury or direct parenchymal injury [29]. The vascular hypothesis of late delayed radiation injury supposes that radiation induces structural changes in the vasculature (vessel wall thickening, wall dilatation, and endothelial cell nuclear enlargement), which in turn leads to ischaemia and white matter necrosis. In addition, animal models have shown that there is a quantitative decrease in vessel density after radiation [30], which in turn increases the risk for white matter ischaemia. The parenchymal hypothesis of late delayed radiation injury supposes that radiation induces direct injury to various parenchymal cell lines, in particular oligodendrocytes, astrocytes, microglia, and neurons. Shinohara et al. demonstrated that radiation induces loss of the oligodendrocyte type-2 astrocyte (O-2A) progenitor cells, preventing the formation of mature oligodendrocytes and ultimately resulting in demyelination and white matter necrosis [31]. Hwang et al. demonstrated that radiation induces microglial activation, which in turns induces astrocyte expression of prostaglandin E_2_ (PGE_2_), stimulating gliosis, and brain oedema [32]. Rosi et al. demonstrated that hippocampal neurons in mice exposed to radiation significantly decreased expression of activity-regulated cytoskeleton-associated (Arc) protein resulting in neuronal dysfunction and suspected cognitive impairment [33]. Although not well understood, it is reasonable to surmise that late delayed radiation injury actually results from a complex interaction between both vascular and parenchymal dysfunction, although more study is needed to further define this dynamic process.

Although it is helpful to understand the temporal relationship of radiation and the pathophysiology of brain injury, many find it more helpful to classify radiation-induced brain injury by the pattern visualised on imaging. What follows is an imaging-based discussion of radiation-induced complications organised by which anatomic structures are affected.

### Radiation-induced vascular injury

As previously stated, radiation can have profound effects on the intracranial vasculature through irreversible endothelial injury. This is typically a late delayed injury manifesting months to years after radiation exposure. On imaging, radiation-induced vascular injury produces three main appearances: radiation-induced vasculopathy, radiation-induced vascular proliferative lesions, and radiation-induced mineralising microangiopathy.

**Fig. 11:**
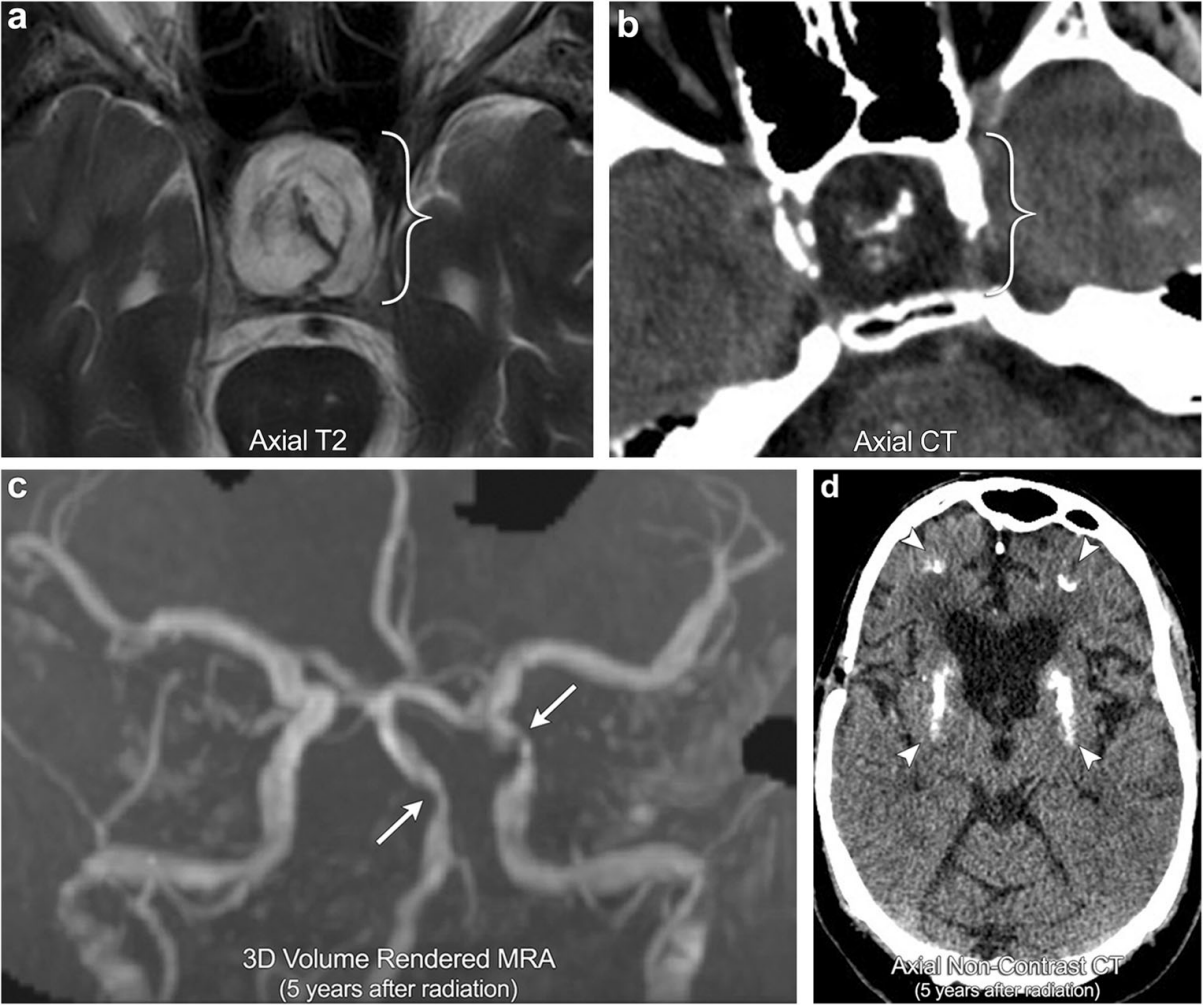
**Radiation-induced vasculopathy and mineralising microangiopathy**. A 35-year-old male with a history of radiation for craniopharyngioma (brackets). The patient went on to develop multiple strokes over subsequent years with MRA images demonstrating multiple areas of severe stenosis of the intracranial vasculature, most notable in the basilar artery and left supraclinoid ICA (arrows); this is compatible with radiation-induced vasculopathy. CT demonstrates dense calcifications in the subcortical white matter and basal ganglia (arrowheads), consistent with mineralising microangiopathy

Radiation-induced vasculopathy refers to accelerated myointimal proliferation of vessel walls resulting in varying degrees of stenosis or occlusion [34]. This process has a tendency to affect the large basal cerebral arteries, often escaping detection on the arteriole level. On angiography, this is typically easy to identify as the affected vessels will be located within the radiation port. For central tumours, such as optic pathway gliomas or sellar lesions, the supraclinoid internal carotid arteries and proximal circle of Willis are particularly vulnerable, often producing a moyamoya imaging pattern (Fig. 11). Important to note, these patients are at increased risk for ischaemia and recurrent infarctions.

**Fig. 12:**
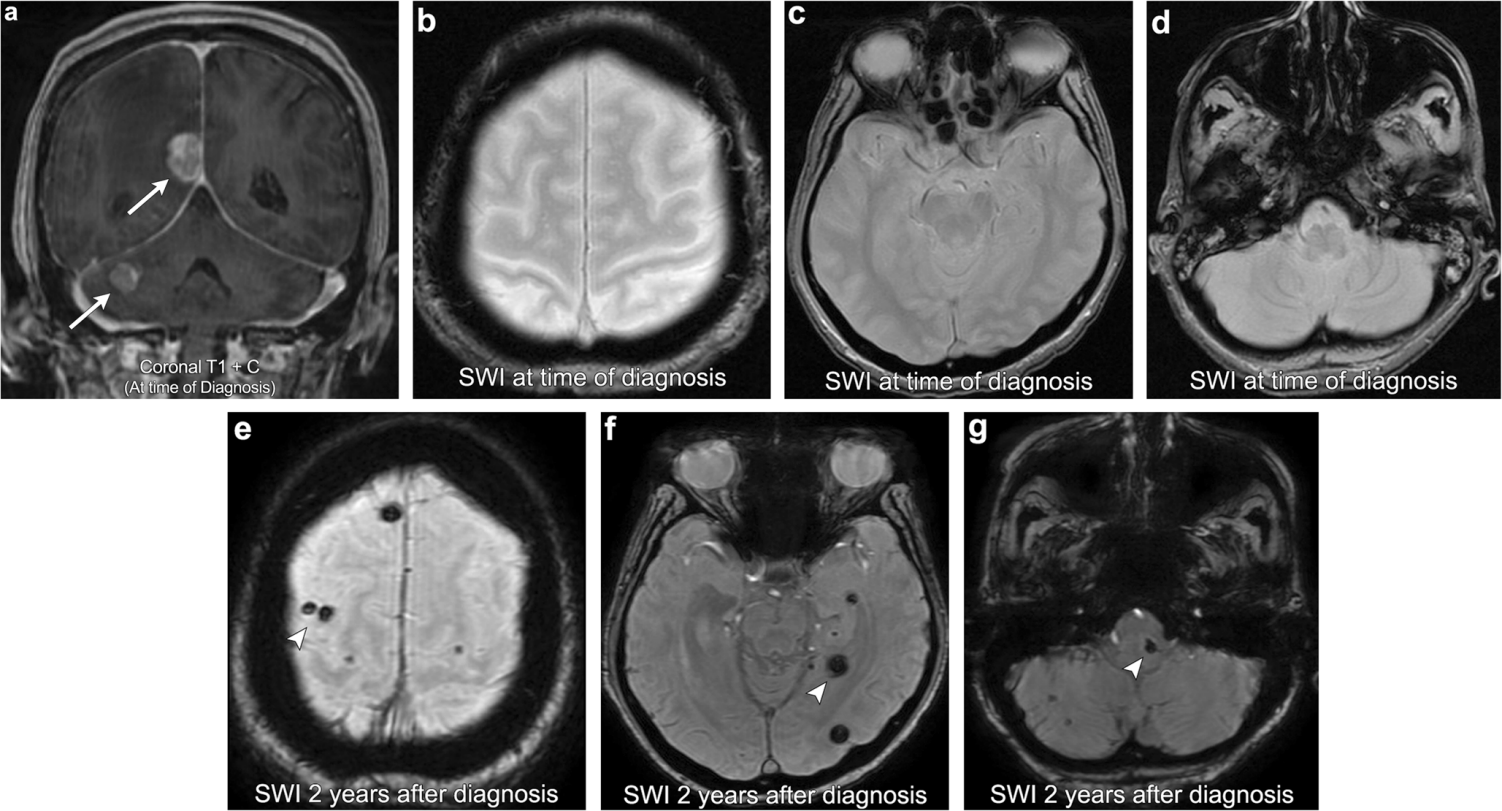
**Radiation-induced cavernous angiomas (cavernomas**). A 76-year-old female with a history of lung cancer, multiple intracranial metastases (arrows). Susceptibility-weighted imaging (SWI) at the time of diagnosis demonstrates no foci of abnormal low T2-star signal/blooming in the brain parenchyma. SWI imaging performed 2 years after whole-brain radiation therapy (WBRT) demonstrates multiple foci of new abnormal low T2-star signal scattered within the brain parenchyma (arrowheads), consistent with radiation-induced cavernous angiomas

Radiation-induced vascular proliferative lesions include capillary telangiectasia and cavernous malformations (cavernomas). Formation of these lesions is thought to result from injury to the cerebral microcirculation, triggering focal regions of neoangiogenesis within the brain parenchyma [35]. Larson et al. even hypothesised that these two lesions exist on the same pathological spectrum with radiation triggering a proliferative pathway that results in capillary telangiectasia forming into cavernomas [36]. Histopathologically, capillary telangiectasia consists of thin-walled ectatic capillaries with normal brain parenchyma dispersed between the vascular channels. On imaging, these lesions demonstrate faint stipple T2 hyperintensity and a blush of enhancement [37]. Susceptibility-weighted imaging (SWI) can also be helpful in making the diagnosis as these lesions demonstrate low signal due to slow flow and increased quantities of deoxyhaemoglobin [37, 38]. In contrast to capillary telangiectasia, cavernomas are comprised of tightly packed immature blood vessels without intervening brain parenchyma. On imaging, these lesions have a propensity for microhaemorrhage producing a mixed T1/T2 signal lesion (“popcorn appearance”) with a haemosiderin lining that blooms on GRE/SWI (Fig. 12), [39].

Radiation-induced mineralising microangiopathy refers to the development of dystrophic microcalcifications in the brain parenchyma. Histopathologically, this process consists of calcium deposition within damaged vessel walls as well as surrounding necrotic brain tissue [40]. On imaging, calcification is typically seen in the basal ganglia and subcortical white matter, likely reflecting the inherit vulnerability that small perforating and peripheral vessels have to radiation injury (Fig. 11).

### Radiation-induced parenchymal injury

**Fig. 13:**
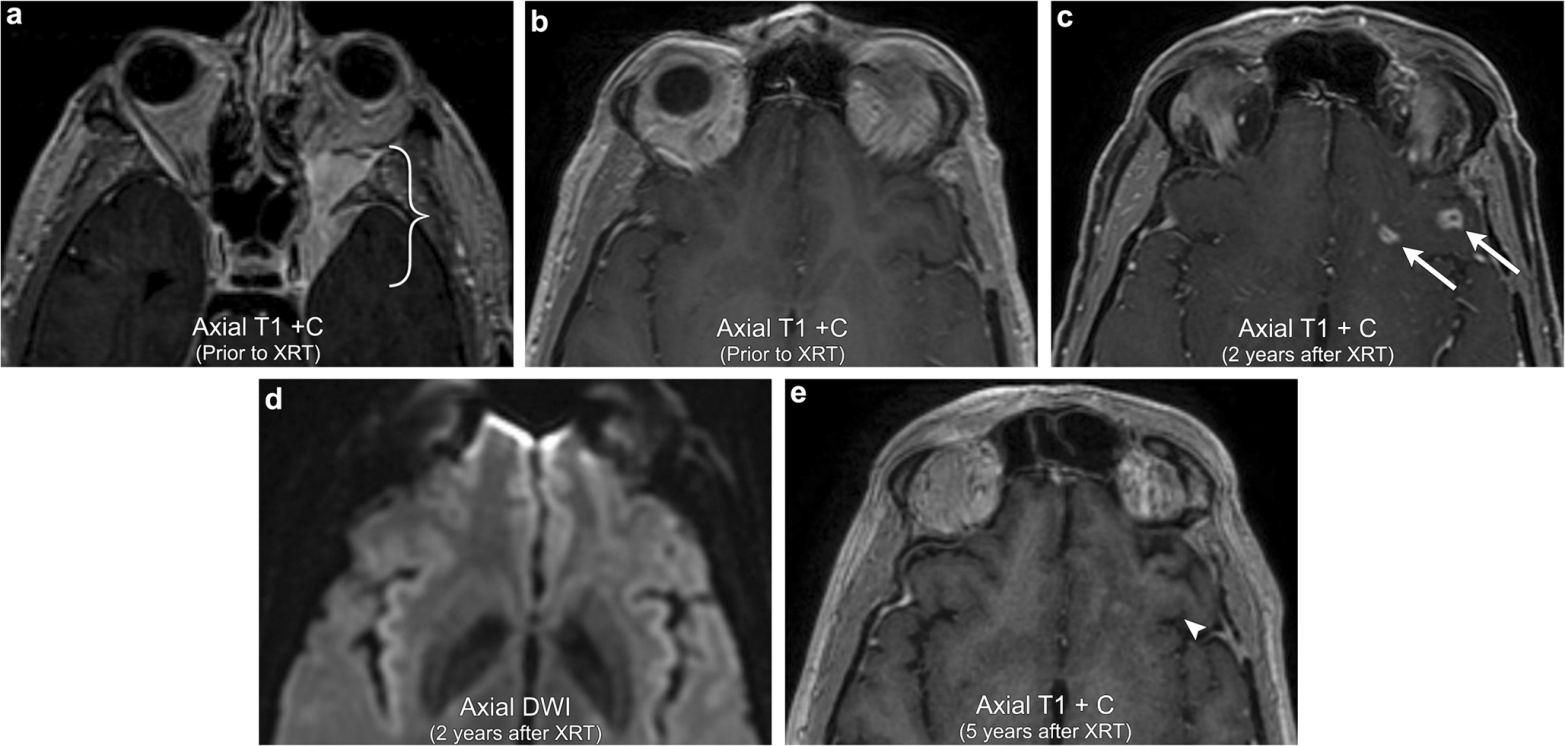
**Radiation necrosis**. A 58-year-old male with left sphenoid wing meningioma extending into the left cavernous sinus and orbital apex (bracket). The left basifrontal region at that time was unremarkable. Follow-up imaging 2 years after receiving radiation now demonstrates several irregular rim-enhancing lesions in the left basifrontal region (arrows). There is no abnormal corresponding DWI signal. Follow-up imaging 5 years after radiation now demonstrates resolution of the irregular enhancing lesions in the left basifrontal region (arrowhead), confirming radiation necrosis

Radiation necrosis, as the name suggests, refers to a combination of vascular and parenchymal injury resulting in necrosis of either tumour or normal brain parenchyma. It is a delayed phenomenon that has a propensity for affecting the white matter and the deep laminae of the overlying cortex with relative sparing of the superficial cortex [41]. The likelihood of radiation necrosis depends on two main factors. The first is the time elapsed since radiation exposure, with 85% of cases occurring within 2 years after radiation. In fact, Shah et al. suggested that any new or worsening abnormality discovered 3 years after completion of radiation is unlikely to represent pure radiation necrosis [42]. The second is the radiation dose, with the prevalence doubling with radiation doses that exceed 62 Gy and quadruples with radiation doses that exceed 78 Gy [42]. On imaging, it can be very difficult to differentiate tumour from radiation necrosis and close-interval follow-up studies are often needed to see if enhancement worsens or improves. That being said, there are three main imaging patterns that are highly suggestive of radiation parenchymal injury. These include (1) new enhancement along the margin of the resection cavity with an internal “soap-bubble appearance”, typically involving tissue that was previously non-enhancing [43], (2) periventricular enhancing foci distant from the resection cavity but within the radiation port, and (3) enhancing foci distant from the resection cavity within the radiation port but not along expected pathways of tumour spread. In all scenarios, advanced MRI imaging can be helpful to discriminate viable tumour from radiation necrosis in that the region of enhancement typically demonstrates decreased rCBV on perfusion imaging, elevated lipid/lactate peaks on MR spectroscopy, and resolution of enhancement on serial follow-up imaging (Figs. 7 and 13).

**Fig. 14:**
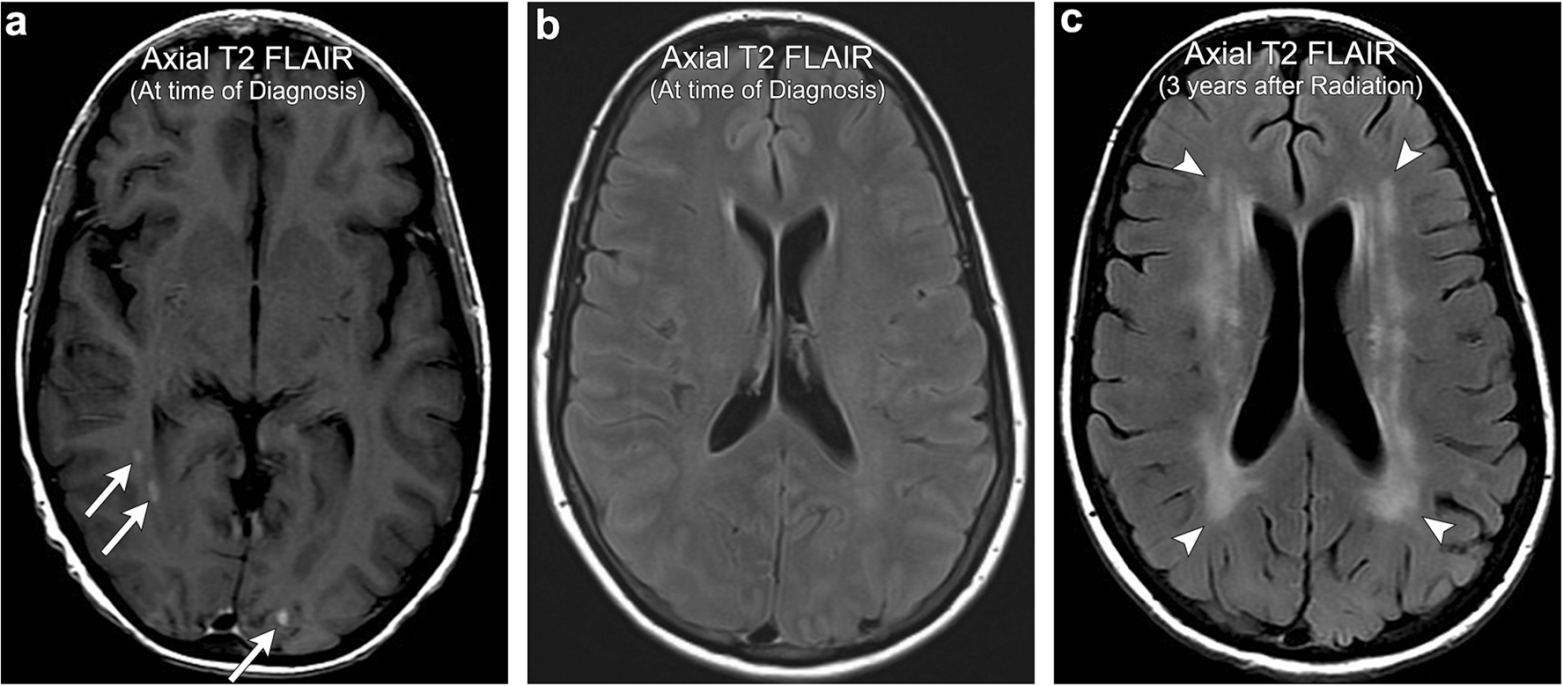
**Radiation-induced leukoencephalopathy**. A 47-year-old female with a history of multiple intracranial thyroid carcinoma metastases (arrows). The patient received WBRT and then developed gradual cognitive decline over the subsequent few years. Imaging performed 3 years after WBRT demonstrates extensive periventricular T2 FLAIR hyperintensity (arrowheads), more than would be expected for age-related microvascular disease, consistent with radiation-induced leukoencephalopathy. Note the relative sparing of subcortical U fibres

Radiation-induced leukoencephalopathy is a term used to denote radiation-induced white matter injury, typically without frank necrosis. As with most forms of radiation injury, the pathophysiology is unclear but appears to result from either direct axonal injury or secondary white matter injury from vascular compromise. The incidence of leukoencephalopathy is also unclear; however, a retrospective study reported an incidence of 34% in patients who received whole-brain radiation treatment (WBRT) for brain metastases after 6 months of follow-up [44]. Clinically, patients present with varying degrees of neurocognitive decline, with many asymptomatic at the time of imaging diagnosis. In fact, the relationship between white matter changes seen on imaging and symptom severity is unclear as many studies have shown a poor correlation with the degree of cognitive decline [45]. On imaging, radiation-induced leukoencephalopathy appears as progressive, symmetric, confluent T2 hyperintensity involving predominantly the periventricular white matter [40]. It is important to note that some brain tumour patients may also have superimposed medication-induced immunosuppression and therefore are also at risk for developing progressive multifocal leukoencephalopathy (PML). Although quite rare, this has been described in several case reports throughout the literature and it is important that the imager be able to discriminate the two white matter entities [46]. In radiation-induced leukoencephalopathy, unlike with PML, white matter changes are more diffuse and tend to spare the subcortical U fibres (Fig. 14).

### Stroke-like migraine attacks after radiation therapy (SMART) syndrome

**Fig. 15:**
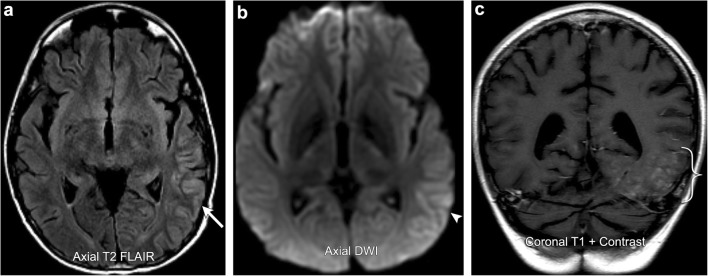
**SMART syndrome**. A 31-year-old male with a history of posterior fossa atypical teratoid rhabdoid tumour resected and radiated 10 years prior, now presents with persistent headaches, visual field deficits, and 1 day of word-finding difficulties. MRI images demonstrate gyriform thickening and a high T2 FLAIR signal involving much of the posterior cortex of the left temporal, parietal, and occipital lobes (arrow). There is associated mild diffusion restriction (arrowhead) and gyriform enhancement (bracket). Follow-up EEG demonstrated no epileptiform activity and symptoms subsequently resolved. Imaging findings and clinical course compatible with SMART syndrome

SMART syndrome is a rare clinical entity that consists of complex migraine-like symptoms in the setting of prior radiation exposure. It is more commonly seen in men (male to female ratio of 2.2:1) and is typically a late delayed phenomenon occurring in patients that received radiation doses of 50–64 Gy [47]. Clinically, these patients present with migranious headaches, often associated with nausea, emesis, and photosensitivity. In addition, 33–74% will have focal neurological deficits and 70–82% will have seizures [47]. The pathogenesis of SMART syndrome is poorly understood and thought to be of multifactorial aetiology. Some authors suggest that radiation-induced vascular injury produces a reversible vascular dysregulation resulting in blood-brain barrier disruption and brain oedema, similar to posterior reversible encephalopathy syndrome (PRES) [48]. However, other authors such as Farid et al. demonstrated normal cerebral vascular reactivity in patients with clinical symptoms of SMART syndrome by comparing ictal and postictal brain perfusion through the use of Tc-99 m HMPAO SPECT, arguing against a vascular mechanism and instead suggesting a component of neuronal dysfunction [49]. On imaging, SMART syndrome classically demonstrates gyriform T2 hyperintensity and enhancement with some cases also showing diffusion restriction. It typically involves the posterior cerebral hemispheres or cerebellum and usually improves or resolves on follow-up imaging (Fig. 15), [48].

## Chemotherapy-related complications

Chemotherapeutic agents can result in direct toxicity to various structures of the central nervous system. The particular CNS structure involved and the degree to which they are affected vary depending on the drug administered and the dose that was given. Given the ever-growing list of new chemotherapeutic agents, each with its own toxic profile, a thorough discussion of CNS drug toxicity is beyond the scope of this article. However, that being said, it has been shown that most drugs tend to affect similar structures and produce similar patterns of injury on imaging. In addition, certain drugs can result in classic pathological imaging findings (i.e., ipilimumab-induced hypophysitis) that are easy to diagnose once familiar with their imaging appearance. What follows is a discussion of these common chemotherapy-related injury patterns.

It has been widely described in the literature that of all the CNS structures, the white matter is particularly vulnerable to drug injury. Although the mechanism through which this occurs is poorly understood, it has been suggested that leukotoxic agents result in disruption of neural transmission, particularly in the neurobehavioural pathways, often resulting in the classic clinical presentation of “altered mental status”. This injury results in a drug-related toxic leukoencephalopathy, producing a clinical and radiographic picture similar to radiation-induced leukoencephalopathy. In fact, many chemotherapeutic agents can actually potentiate many of the radiation injuries previously discussed. The most common leukotoxic agent used in clinical practice is methotrexate; however, many other agents including carmustine, cisplatin, cytaribine, fluorouracil, and interleukin-2 have also been implicated [50, 51]. The incidence with which neurotoxicity occurs is quite variable; however, the route of administration has been shown to be important. Filley et al. stated that toxic leukoencephalopathy may occur in less than 10% treated with intravenous methotrexate, but in up to 40% treated with intrathecal methotrexate [50]. On imaging, chemotherapy-related leukoencephalopathy typically produces non-enhancing T2 hyperintensity in the frontoparietal white matter, but can sometimes demonstrate diffuse involvement of the periventricular and deep white matter, similar to radiation-induced leukoencephalopathy [51]. On DWI, focal or diffuse areas of reversible restricted diffusion may be seen in acute cases and typically show improvement over time after cessation of the offending drug [52].

**Fig. 16:**
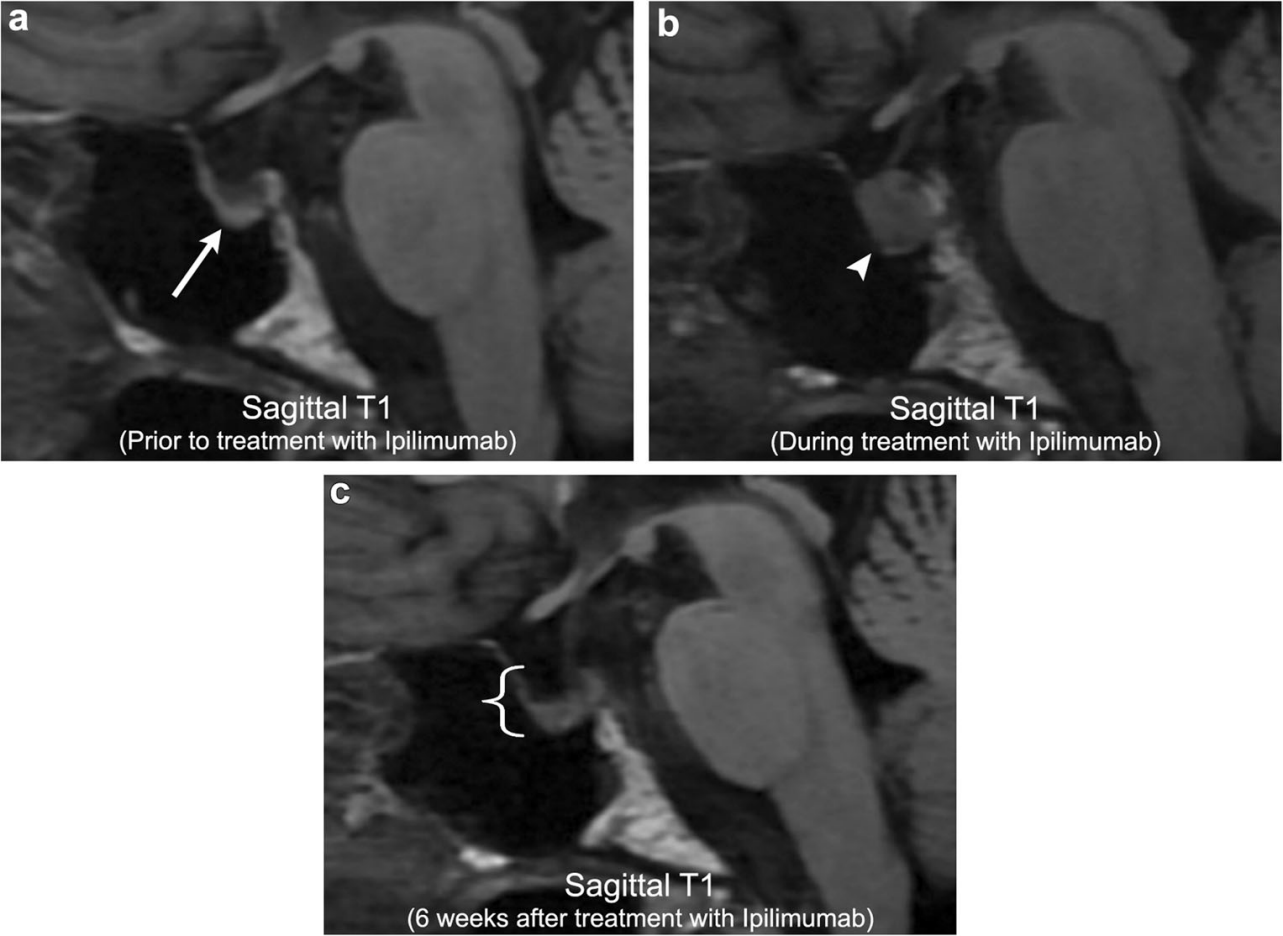
**Ipilimumab-induced hypophysitis**. A 63-year-old male with metastatic melanoma. Initial imaging demonstrates a partially empty sella with an otherwise normal pituitary gland (arrow). After initiation of ipilimumab, there is marked heterogeneous enlargement of the pituitary gland (arrowhead) compatible with ipilimumab-induced hypophysitis. Imaging 6 weeks after cessation of ipilimumab demonstrates resolution of pituitary enlargement (bracket). However, patient now has clinical and laboratory evidence of panhypopituitarism

Another commonly discussed chemotherapy-related complication is that of ipilimumab hypophysitis. Ipilimumab (MDX-010) is a monoclonal antibody that works by suppressing cytotoxic T lymphocyte-associated antigen 4, which in turn augments the patient’s immune system to mount a response against malignant cells. This agent has become particularly effective for metastatic melanoma and renal cell carcinoma, where it is now widely used for patients with disseminated disease. However, augmentation of the immune response can result in many adverse autoimmune reactions throughout the body, such as colitis, dermatitis, and arthritis, to name a few. In the CNS, ipilimumab-induced hypophysitis is one of the better recognised autoimmune reactions described in the literature. Although this is thought to occur in < 5% of all ipilimumab-treated patients, these patients can clinically present with life-threatening hormonal imbalance, particularly hypercortisolism. On imaging, ipilimumab-induced hypophysitis results in diffuse enlargement of the pituitary gland, often hypointense on T1-weighted imaging, with variable thickening/enlargement of the infundibulum [53, 54]. Follow-up imaging after cessation of ipilimumab and introduction of steroids typically demonstrates complete resolution of abnormal findings with return of the pituitary gland to its baseline appearance (Fig. 16). This imaging resolution will coincide with resolution of clinical symptoms and changes in hormone levels. Importantly, many patients will go on to develop varying degrees of hypopituitarism, necessitating hormone replacement.

## Surgery-related complications

**Fig. 17:**
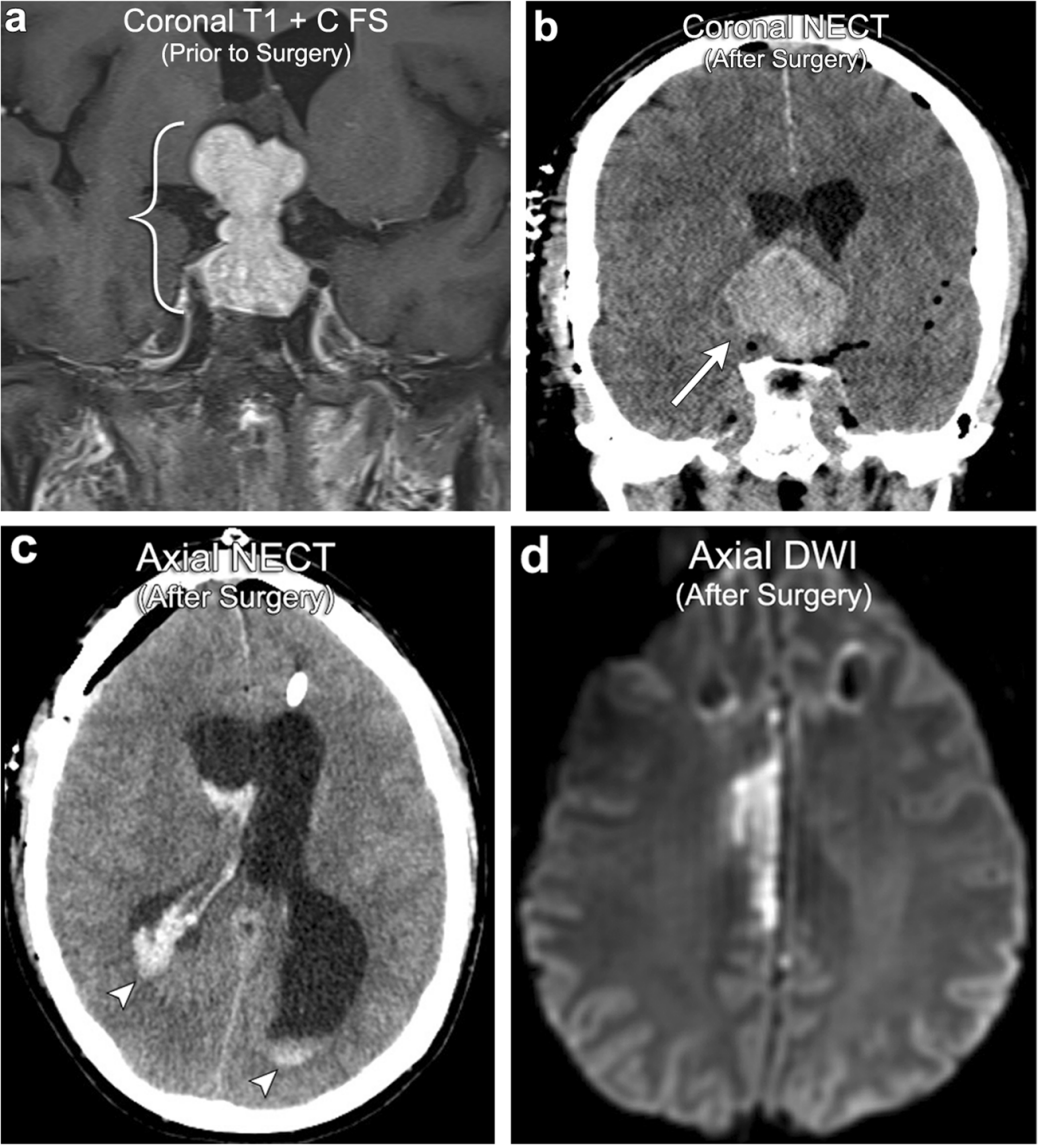
**Postoperative complication: haemorrhage and infarct**. A 52-year-old male with a large pituitary macroadenoma (bracket). Postoperative imaging after gross resection demonstrates haemorrhagic enlargement of the residual component of the adenoma consistent with apoplexy (arrow). There is also intraventricular extension of haemorrhage (arrowheads). Follow-up MRI demonstrates right anterior cerebral artery (ACA) territory infarction due to mass effect on the right ACA from the enlarged haemorrhagic pituitary adenoma

**Fig. 18:**
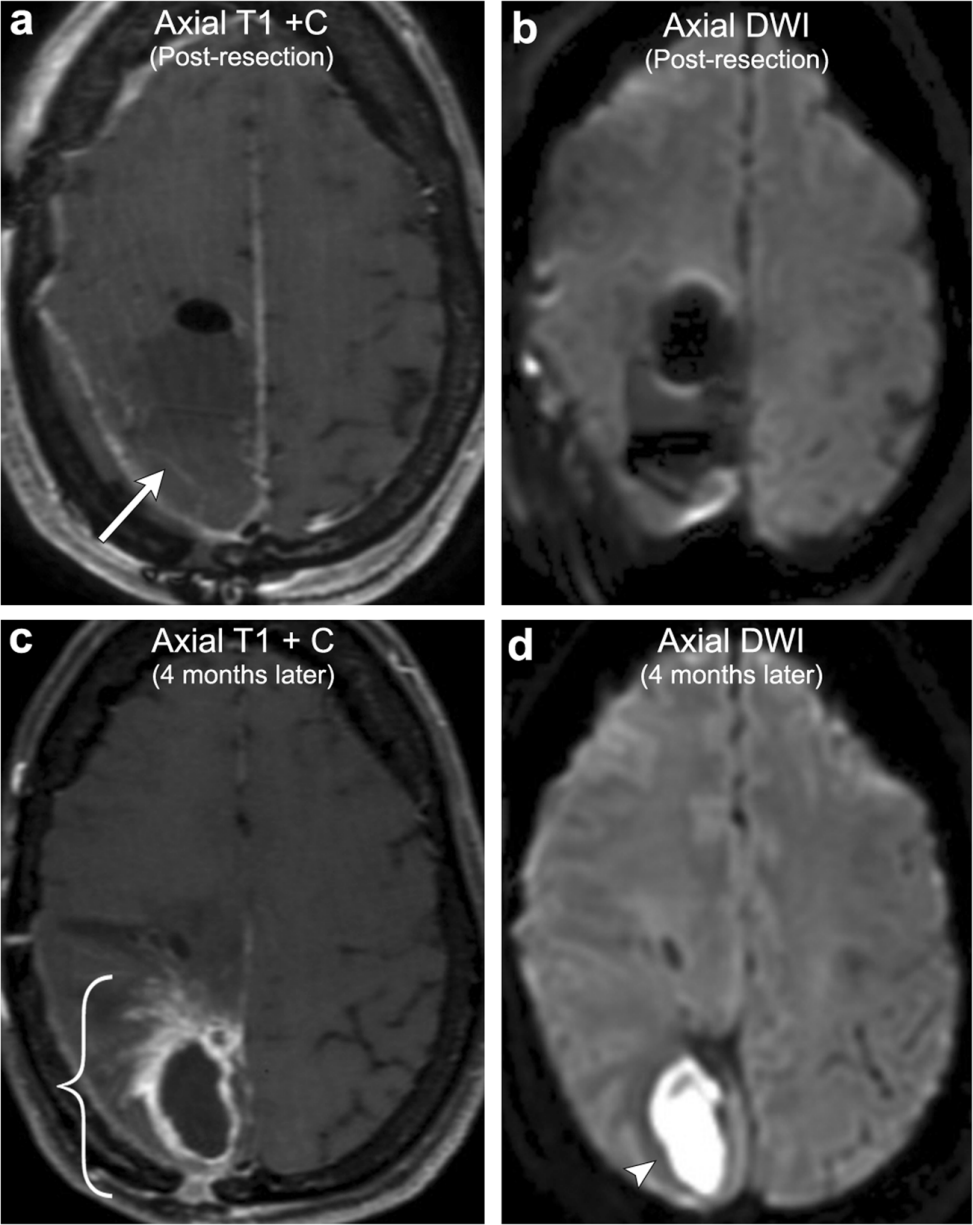
**Postoperative complication: abscess**. A 45-year-old female with a history of right parietal lobe anaplastic astrocytoma resection. Initial post-resection imaging demonstrates faint peripheral enhancement of the surgical cavity (arrow) and no internal diffusion restriction. About 4 months after, the patient described a persistent “scab” over the surgical site, increasing headaches, and subjective fever. Repeat imaging shows thick rim enhancement (bracket) with internal diffusion restriction (arrowhead), consistent with abscess formation, confirmed intraoperatively

Surgical resection is a key component of treating many types of brain tumours, in particular, high-grade gliomas, large metastases, or extra-axial lesions with significant mass effect. Although usually uneventful, surgery does carry its own risks, with one study demonstrating a 3.4% risk of having at least one surgical complication in patients undergoing surgery for malignant brain tumours [55]. The most common post-resection complication is iatrogenic stroke, with an estimated incidence of 1.6%. Iatrogenic stroke carries a nine-fold increased risk of in-hospital mortality and the risk of developing ischaemic lesions is correlated to the proximity of the tumour to central perforating arteries [56] (Fig. 17). The second most common post-resection complication is intracranial haemorrhage, with an estimated incidence of 1.0% (Fig. 17). Intracranial haemorrhage carries a three-fold increased risk of in-hospital mortality and may require repeat surgery depending on the extent of haemorrhage. As compared with infarct and haemorrhage, post-resection infection is a less common rare complication typically seen in patients with a depressed immune status. Manifestations include bone flap infection, subdural empyema, cerebritis, abscess (Fig. 18), and meningitis (incidence of approximately 0.1%). Although relatively rare, early recognition of these postoperative complications is extremely important in order to avoid significant morbidity/mortality.

## Conclusion

Primary and metastatic brain tumours are frequently encountered in the daily practice of neuroimaging. The wide array of treatment options currently available to treat these tumours has made interpretation of post-treatment imaging quite complex. Knowledge of post-treatment imaging techniques, treatment response criteria, and commonly encountered treatment-related complications will simplify the approach to this challenging topic.
